# Is reverse total shoulder arthroplasty (rTSA) more advantageous than anatomic TSA (aTSA) for osteoarthritis with intact cuff tendon? A systematic review and meta-analysis

**DOI:** 10.1186/s10195-022-00625-y

**Published:** 2022-01-06

**Authors:** Hyojune Kim, Chul-Ho Kim, Minsoo Kim, Wonsun Lee, In-Ho Jeon, Kwang Won Lee, Kyoung Hwan Koh

**Affiliations:** 1 Department of Orthopaedic Surgery, Daejeon Eulji Medical Center, Eulji University School of Medicine, Daejeon, Republic of Korea; 2 Department of Orthopaedic Surgery, Asan Medical Center, University of Ulsan College of Medicine, 88 Olympic-ro 43-gil, Songpa-gu, Seoul, 05535 Republic of Korea; 3 Department of Orthopaedic Surgery, Chung-Ang University Hospital, Chung-Ang University College of Medicine, Seoul, Republic of Korea

**Keywords:** Anatomical total shoulder arthroplasty, Reverse total shoulder arthroplasty, Range of motion, Complications, Revision rate

## Abstract

**Purpose:**

We aimed to compare the outcomes and complications of anatomical shoulder arthroplasty (aTSA) and reverse total shoulder arthroplasty (rTSA) for primary glenohumeral osteoarthritis with intact cuff tissue.

**Materials and methods:**

The MEDLINE, Embase, and Cochrane Library databases were systematically searched for studies published before March 2, 2021 using the PRISMA guidelines. Studies were included if they directly compared aTSA and rTSA for treating primary glenohumeral arthritis. A meta-analysis was performed using six studies that compared radiologic outcomes, functional scores, and range of motion (ROM). All the data were pooled using a random-effects model. Odds ratios (ORs) and 95% confidence intervals (CIs) were calculated as dichotomous data, while continuous data were analyzed using mean differences with 95% CIs.

**Results:**

Two independent researchers reviewed 1,061 studies. Six studies met the inclusion criteria. The range of motion, especially external rotation, was better for aTSA than for rTSA (MD = − 10.28, 95% CI: − 16.69 to − 3.88, *P* = 0.002). Functional scores showed no difference between aTSA and rTSA. Glenoid loosening (OR = 0.17, 95% CI: 0.06–0.50, *P* = 0.001) was more common with aTSA, and scapula notching (OR = 10.63, 95% CI: 1.73–65.27, *P* = 0.01) with rTSA. In the mid-term follow-up, the overall revision rate showed no difference between aTSA and rTSA, with a pooled OR of 0.33 (95% CI: 0.07–1.57, *P* = 0.16).

**Conclusion:**

A better ROM was achieved after aTSA than after rTSA. There was no difference in the revision rate at mid-term follow-up between aTSA and rTSA. Glenoid loosening was more common with aTSA, and scapula notching with rTSA.

*Level of evidence:* Level IV, Meta-analysis.

## Introduction

Anatomical total shoulder arthroplasty (aTSA) and reverse total shoulder arthroplasty (rTSA) are commonly performed in older patients. The decision to carry out aTSA is dependent on the continuity of the rotator cuff tissue. Previous studies have shown that patients with cuff tears may experience poorer outcomes than those without cuff tears do after aTSA because of increased instability caused by the proximal migration of the humeral head and the loosening of the glenoid component [3]. Moreover, one of the most common causes of revision after aTSA is cuff tear [18]. Therefore, rTSA, traditionally performed for cuff tear arthropathy, is increasingly being used as a first-line option, instead of aTSA, for older patients with primary glenohumeral osteoarthritis with intact cuff tissue because of concerns about postoperative tear [13, 28]. In addition, if aTSA is performed in cases of severe glenoid wear, severe edge loading or posterior subluxation can occur because of glenoid retroversion [21]. This could be another reason for choosing rTSA over aTSA.

Previous studies have compared the outcomes of rTSA and aTSA. In their meta-analysis published in 2017, Bohsali et al. found that aTSA was associated with slightly fewer complications than rTSA was (6.6 vs. 7.3%) [3]. Although early reports have shown higher rates of complications with rTSA than with aTSA [4, 6, 25], the complication rates have decreased thanks to advanced implant designs and the expertise of surgeons. Therefore, in the most recent study that used a large prospective database, primary aTSA led to significantly greater complication and revision rates than rTSA did [18]. However, there were different surgical indications for aTSA and rTSA. Primary osteoarthritis was the most common indication for aTSA, and cuff tear arthropathy was the most important reason for rTSA. To our knowledge, no study has directly compared the outcomes of the two surgical options in the case of primary osteoarthritis with intact cuff.

Therefore, the goal of this study was to compare the outcomes and complications of aTSA and rTSA for treating primary glenohumeral osteoarthritis with intact cuff tissue. We hypothesized that aTSA led to better clinical outcomes in terms of range of motion (ROM) and functional scores and a lower revision rate than rTSA.

## Materials and methods

The present study was performed in accordance with the guidelines of Cochrane Reviews and the Preferred Reporting Items for Systematic Review and Meta-Analysis Protocols guidelines [11, 16]. Although the present study involved human participants, ethical approval or informed consent from the participants was not required because all the data were obtained from previously published studies that were analyzed anonymously without causing any potential harm to the participants.

### Literature search

Using the aforementioned guidelines, the following comprehensive literature databases were searched for studies that compared aTSA with rTSA for the treatment of primary glenohumeral arthritis with intact rotator cuff: MEDLINE, Embase, and the Cochrane Library. The search identified articles published up to March 2, 2021 using an a priori search strategy. The following search terms were used: (“aTSA” OR “TSA” OR “total shoulder arthroplasty” OR “total shoulder replacement” OR “shoulder arthroplasty”) AND (“rTSA” OR “RTSA” OR “reverse arthroplasty” OR “reverse shoulder arthroplasty” OR “reverse total shoulder arthroplasty” OR “reverse shoulder replacement”) AND (“shoulder OR glenohumeral”) AND (“arthritis OR osteoarthritis”). There were no restrictions on language or publication year. After the initial electronic search, relevant articles and their bibliographies were manually searched.

### Study selection

From the obtained titles and abstracts of the studies, two board-certified orthopedic surgeons with an orthopedic shoulder fellowship independently selected the studies for full-text review. If the abstract provided insufficient data to make a decision, the full article was reviewed.

Studies were included in the systematic review if (1) they directly compared rTSA and aTSA and (2) they reported complete data, including means, standard deviations, sample sizes, and percentages. We only included original research articles. Biomechanical and cadaveric studies, technical notes, letters to the editor, expert opinions, review articles, meta-analyses, conference abstracts, and case reports were excluded. We also excluded (1) studies in which aTSA or rTSA was performed for other reasons such as cuff tear arthropathy, secondary osteoarthritis by trauma, infection, and instability; (2) studies that included patients who underwent revision surgery; and (3) studies that investigated the same patient groups involved in previously published studies (i.e., each participant cohort could only be included once in this systematic review).

At each stage of study selection, the *κ* value was calculated to determine the inter-reviewer agreement regarding study selection. Agreement between the reviewers was correlated a priori using the following *κ* values: *κ* = 1 corresponded to “perfect” agreement; 1.0 > * κ* ≥ 0.8 to “almost perfect” agreement; 0.8  >* κ* ≥ 0.6 to “substantial” agreement; 0.6  > * κ* ≥ 0.4 to “moderate” agreement; 0.4  >* κ* ≥ 0.2 to “fair” agreement; and * κ* < 0.2 to “slight” agreement. Disagreements at each stage were resolved by consensus between the two investigators or by discussion with a third investigator, who was a board-certified orthopedic surgeon.

### Data extraction

To analyze the qualitative data, the following information and variables were extracted using a standardized form: (1) study design, (2) number of patients investigated, (3) mean patient age, (4) procedure (rTSA or aTSA), (5) prosthesis, (6) follow-up period, and (7) outcome measures.

In the pooled analysis, the following data related to rTSA or aTSA were extracted from the included studies: (1) radiological outcomes (glenoid loosening and scapular notching), (2) revision rate, (3) functional scores (subjective shoulder value; Constant score; simple shoulder test; and the American Shoulder and Elbow Surgeons Score, ASES), and (4) range of motion (forward elevation, external rotation, abduction, and internal rotation).

The same two board-certified orthopedic surgeons who participated in the study selection independently extracted and recorded the data from each enrolled study. Disagreements between the reviewers were resolved by a discussion.

### Methodological quality assessment

The methodological quality of the included studies was assessed using the Methodological Index for Nonrandomized Studies (MINORS) [22], which is a valid tool for assessing the quality of both randomized controlled trials (RCTs) and non-randomized studies. The maximum MINORS checklist score for comparative studies is 24. Two independent reviewers performed the quality assessment and resolved disagreements through a discussion.

### Data and statistical analyses

The main outcome of the present meta-analysis was a comparison of the degree of ROM between patients who underwent rTSA and aTSA, including the details of forward elevation (FE), external rotation (ER), abduction, and internal rotation (IR). The secondary outcomes were the functional scores (subjective shoulder value, SSV (%); Constant score; simple shoulder test, SST; and ASES) and radiologic complications (glenoid loosening and scapular notching). Additionally, we performed pooled analyses of the revision rates.

For all comparisons, odds ratios (ORs) and 95% confidence intervals (CIs) were calculated as dichotomous data, while continuous data were analyzed using mean differences (MDs) with 95% CIs. Heterogeneity was assessed using the *I*^2^ statistic, where 25, 50, and 75% were considered low, moderate, and high heterogeneity, respectively. Forest plots were used to show the outcomes, pooled estimates of effects, and the overall summary effect in each study. Statistical significance was set at *P* < 0.05. All the data were pooled using a random-effects model, which was previously recommended as a means to avoid overestimating the study results, especially in the field of medicine [20]. The statistical analyses were performed using Review Manager (RevMan) software (version 5.3; Copenhagen), Nordic Cochrane Center, Cochrane Collaboration 2014.

## Results

### Study identification

**Fig. 1:**
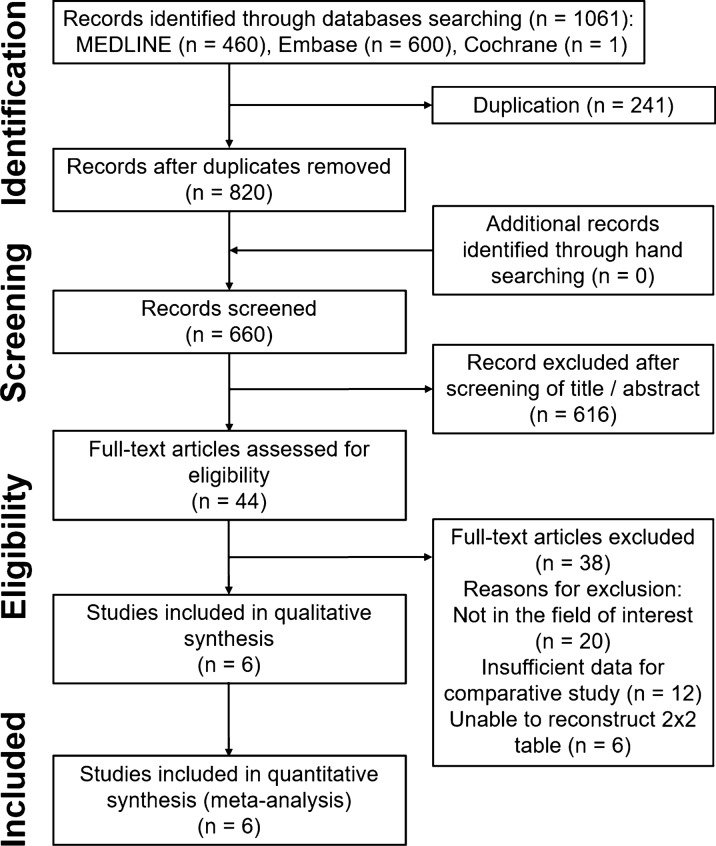
Preferred Reporting Items for Systematic Reviews and Meta-analyses (PRISMA) flow diagram for the identification and selection of studies included in the meta-analysis

The details of the study identification and selection results are summarized in Fig. 1. The initial electronic literature search yielded 1,061 articles. After removing 241 duplicates, 820 studies were screened. Of these, 616 were excluded after screening the titles and abstracts, and 38 were excluded after a full-text review. Thus, six studies [1, 8, 10, 15, 23, 27] were eligible for qualitative and quantitative data analysis. The agreement in study selection between the two reviewers was “substantial” at the title review stage (*κ* = 0.777), “almost perfect” at the abstract review stage (*κ* = 0.837), and “perfect” at the full-text review stage (*κ* = 1.0).

### Study characteristics

All of the six included studies were retrospective cohort studies [1, 8, 10, 15, 23, 27]. In total, 447 cases of glenohumeral arthritis treated with arthroplasty were reported, including 129 treated with rTSA and 318 treated with aTSA. In the included studies, Tornier, DJO, Biomet, Aramis, and Ascend systems were used for rTSA or aTSA. The mean follow-up duration was 24–97 months. The details of each variable are outlined in Table 1.

**Table 1:** Studies included in the meta-analysis

Study	Procedure (cases)	Prosthetic	Age, years	Follow-up time, months ± SD (range)	Outcome measures	Study design	Evidence level	MINORS score
Gallusser et al. [8]	rTSA (8)	Tornier, Edina, MN, USA	79 (73–85)	43 (24–69)	SSV (%), QuickDASH, SST, Constant score, ROM, radiological radiolucent line grading, complications	Retrospective case series	IV	18
	aTSA (19)		66 (47–79)	57 (24–95)				
Steen et al. [23]	rTSA (24)	DJO Surgical, Austin, TX, USA	77.7 ± 8.0	42 (24–92)	ASES, SST, ROM, complications	Retrospective cohort design, treatment study	III	16
	aTSA (96)		76.7 ± 8.0	49 (25–97)				
Alentorn-Geli et al. [1]	rTSA (16)	Biomet, Warsaw, IN, USA	72.5 ± 5.4	35.1 ± 14.2	ROM, PVAS, ASES, SST, patient satisfaction, complications	Retrospective cohort design, treatment study	III	16
	aTSA (15)	Biomet, Warsaw, IN, USA Stryker, Mahwah, NJ, USA Smith and Nephew, Memphis, TN, USA	70.5 ± 7.5	42.7 ± 18.4				
Haritinian et al. [10]	rTSA (12)	Aramis Reversed and Anatomical Total Shoulder Prostheses (3S Ortho, Lyon, France)	71 ± 11	> 24 months	ROM, Constant score, SSV, patient satisfaction	Retrospective cohort design, treatment study	III	16
	aTSA (39)		68 ± 7.5					
Wright et al. [27]	rTSA (33)		78	85	PVAS, patient satisfaction, ASES, WOOS, ROM	Retrospective cohort design, treatment study	III	16
	aTSA (102)		77					
Merolla et al. [15]	rTSA (36)	Ascend Flex (Wright Medical, Memphis, TN, USA)	71.6 (68–72)	28.8 (27–30)	ROM, PVAS, Constant score, radiological outcomes	Retrospective cohort design, treatment study	III	16
	aTSA (47)							

### Methodological quality assessment

The MINORS score for methodological quality assessment was 16.3/24 (range, 16–18) (Table 1). Regarding the eight main evaluation parameters, all six of the included studies lost a point for their retrospective study design and for not clearly describing the assessments (bias) of their endpoints. They also lost a point if the study size was not prospectively calculated [1, 8, 10, 15, 23, 27]. In addition, five studies lost a point because they had a lost-to-follow-up rate of more than 5% of the initial patients [1, 10, 15, 23, 27]. No deductions were made in the additional domains.

### Quantitative data synthesis

#### Range of motion

**Fig. 2:**
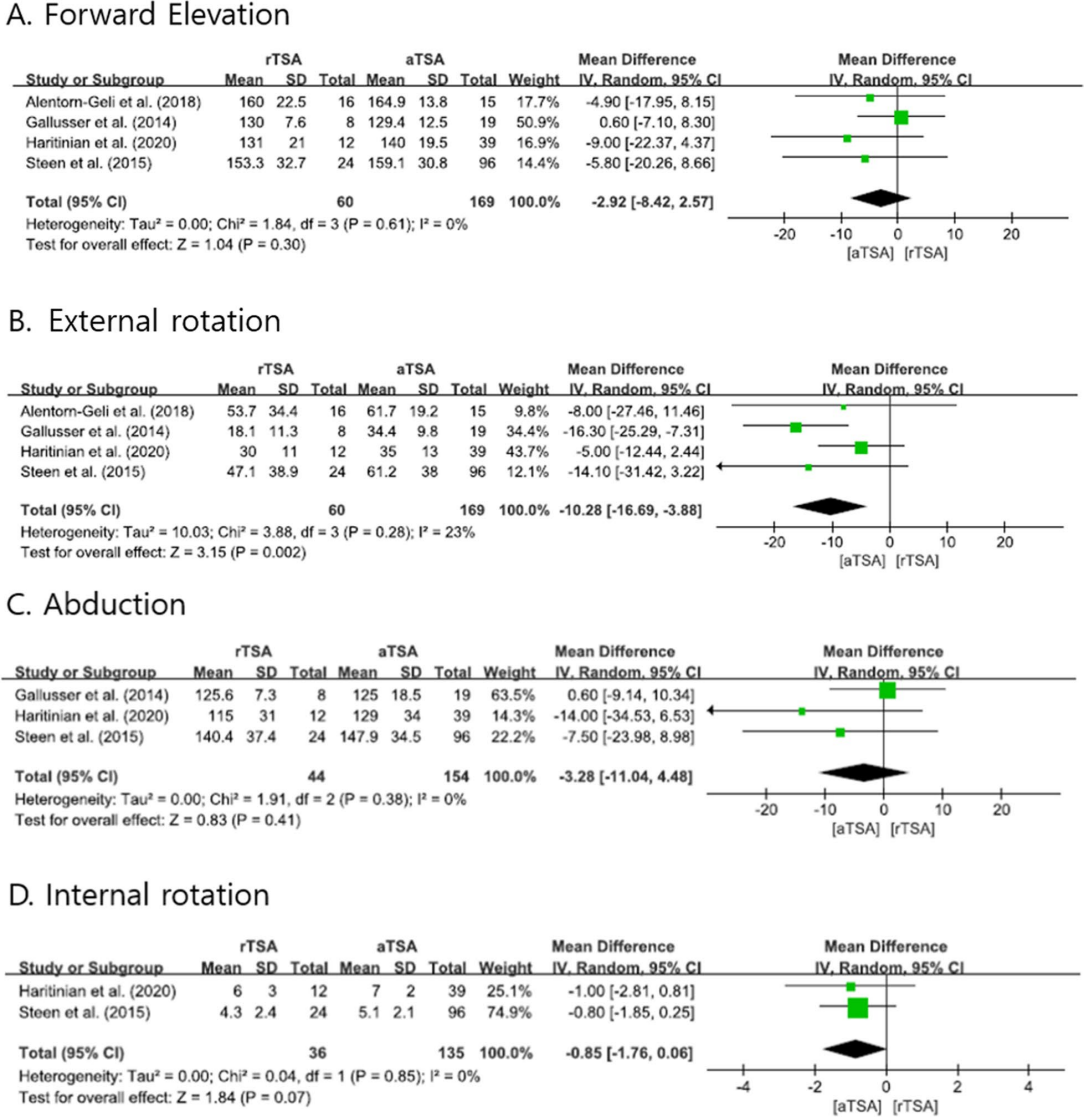
Forest plots showing the differences in range of motion between reverse total shoulder arthroplasty and anatomical total shoulder arthroplasty

Four studies [1, 8, 10, 23] compared the ROM for FE and ER. The pooled analysis showed no difference in the degree of FE between the two arthroplasty systems (mean difference [MD] =  − 2.92, 95% CI: − 8.42–2.57, *P* = 0.30, *I*^2^ = 0%; Fig. 2A). There was a significant difference in the degree of ER (MD = − 10.28, 95% CI: − 16.69 to − 3.88, *P* = 0.002, *I*^2^ = 23%; Fig. 2B). The ROM values for abduction and IR showed no difference between systems (Fig. 2C, D).

#### Functional scores

**Fig. 3:**
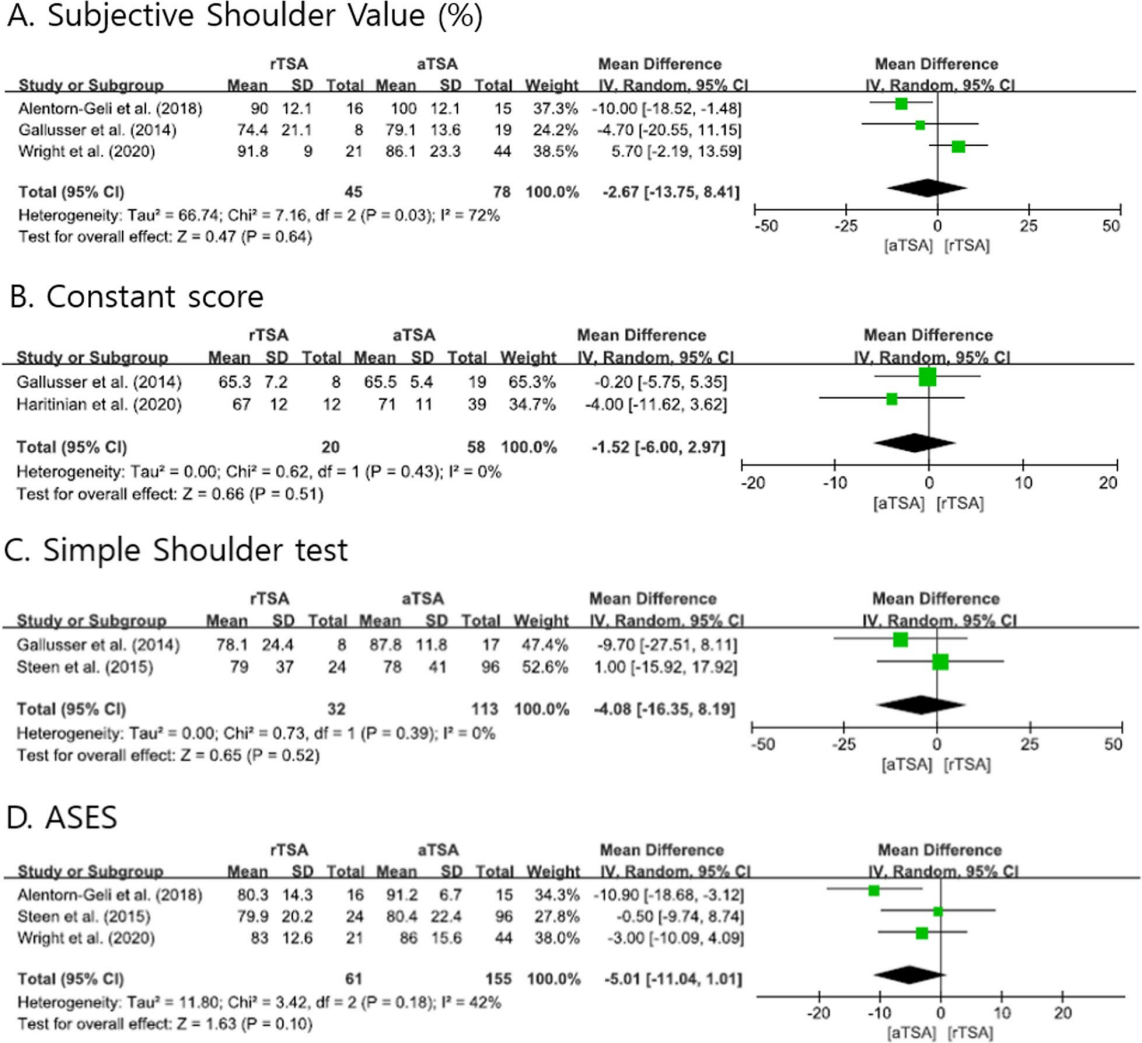
Forest plots showing the differences in functional scores between reverse total shoulder arthroplasty and anatomical total shoulder arthroplasty

The evaluated functional scores, such as SSV, Constant score, SST, and ASES, were pooled and analyzed to compare the two systems; no significant difference was observed. Figure 3 shows forest plots of the differences in functional scores between rTSA and aTSA.

#### Radiologic outcomes and revision rates

**Fig. 4:**
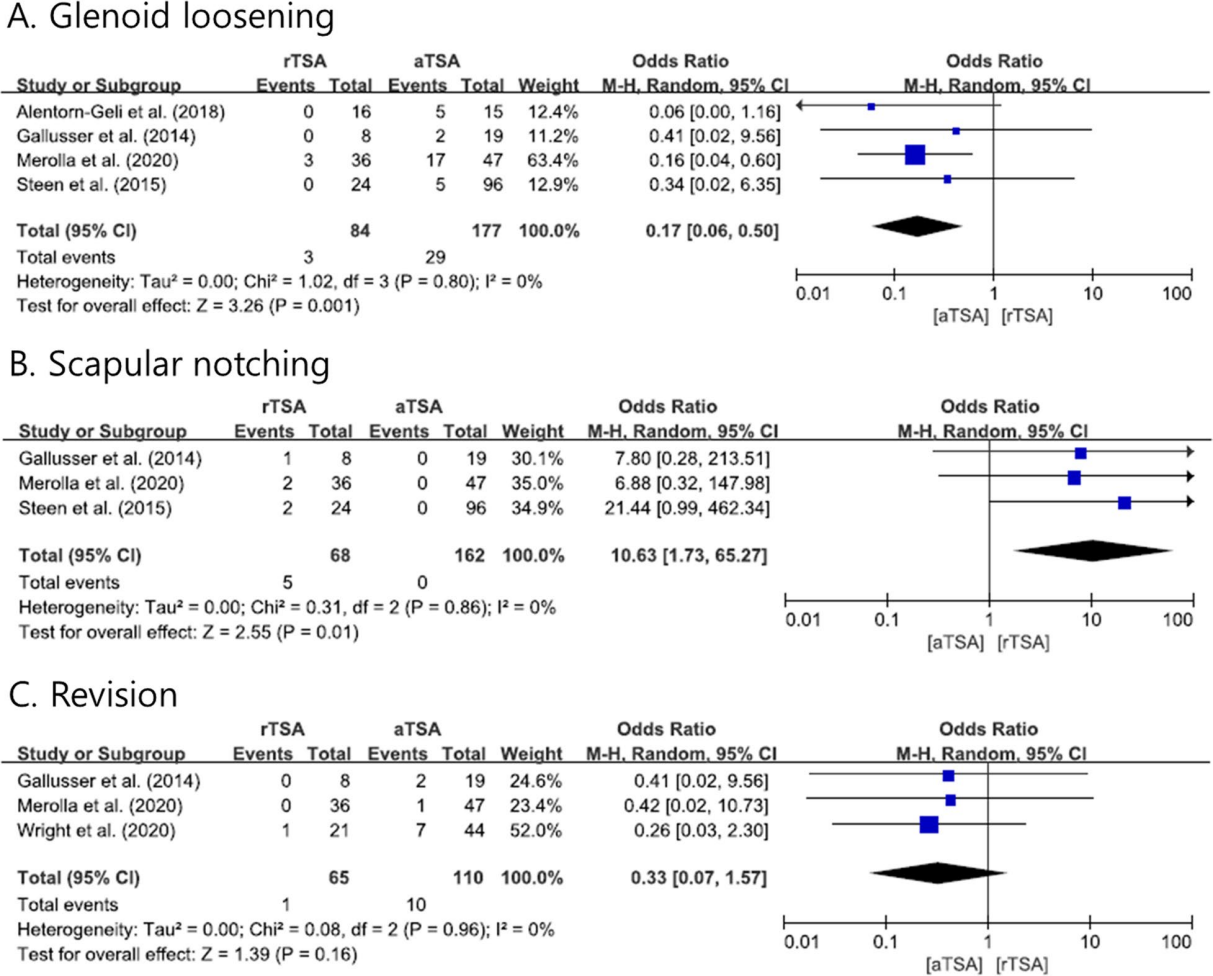
Forest plots showing the odds ratios of complications due to reverse total shoulder arthroplasty and anatomical total shoulder arthroplasty

We extracted data about the radiological changes near the implant in both the rTSA and the aTSA groups. Glenoid loosening and scapular notching were reported in our included studies. In four studies [1, 8, 15, 23], glenoid loosening was lower in the rTSA than in the aTSA group, with a pooled OR of 0.17 (95% CI: 0.06–0.50, *P* = 0.001; Fig. 4A). Scapular notching was higher in the rTSA group than in the aTSA group in three studies [8, 15, 23], with a pooled OR of 10.63 (95% CI: 1.73–65.27, *P* = 0.01; Fig. 4B). Among the 318 patients in the aTSA group, nine patients underwent a revision operation for the following reasons: posterior cuff tear (5), greater tuberosity fracture with rotator cuff dysfunction (1), persistent posterior instability (2), and infection (1). In the rTSA group (129), only one patient underwent revision surgery for infection. The overall revision rate did not differ between systems, with a pooled OR of 0.33 (95% CI: 0.07–1.57, *P* = 0.16; Fig. 4C). The above data show that the heterogeneity was low (*I*^2^ = 0%); forest plots and related details are shown in Fig. 4.

## Discussion

The principal finding of this pooled study was that when arthroplasty was performed in cases of primary osteoarthritis with intact rotator cuff, the ROM was better with aTSA than with rTSA, but the functional scores did not differ between the groups. In addition, radiologically, glenoid loosening was commonly found in the aTSA group and scapular notching in the rTSA group during the follow-up period. However, the overall revision rate was not significantly different between the arthroplasty options. Therefore, we found that aTSA showed better outcomes than rTSA in terms of ROM, but they had similar mean functional scores and revision rates.

Moreover, the ROM of ER was better with aTSA than with rTSA because of the difference in their underlying mechanisms. A previous study, not included in our final analysis, reported that the functional outcome and survival rate during the short-to-medium follow-up period were acceptable, and that there were no significant complications [24]; in most cases, although the FE and ER were limited on the rTSA side compared to the non-affected side, there were no complaints about daily activity. This is similar to our finding of no difference between aTSA and rTSA in terms of functional scores. Perhaps an increased ROM did not directly result in functional improvement, at least in daily activity. However, variables such as age, patient’s activity status, type of work, and correspondence of the affected side to the dominant side should be considered when deciding the treatment option for glenohumeral osteoarthritis with intact cuff tissue.

Another finding of our study was that there was no difference in mean revision rate between the two treatment options. However, most of the studies included in our study had a mid-term follow-up (> 2 years). In addition, the main causes of revision after aTSA were rotator cuff tear and posterior subluxation. Our analysis did not show a statistical difference in the revision rates of rTSA and aTSA; however, an increase in the number of cases and the follow-up period could make a difference. According to our results, rTSA is preferred over aTSA for osteoarthritis without cuff tear as it shows a lower revision rate for patients with poor-quality cuff tissue or a severely worn glenoid. Hence, to select the best arthroplasty system, the condition of the cuff tissue and glenoid wear should be considered.

Regarding radiological outcomes, glenoid loosening is commonly found in cases of aTSA, which is possibly related to the larger ROM obtained with aTSA than with rTSA. Although there was no difference in the revision rate, these changes can have an effect in the long term. When other joints undergo arthroplasty, peri-implant loosening is a warning sign for revision [7, 17, 19]. However, in some studies regarding aTSA, early baseplate loosening found on a radiological exam did not deteriorate in the long-term follow-up [2, 5, 9, 12, 26]. In cases of rTSA, scapular notching did not improve in the long-term follow-up, resulting in a significantly negative effect on the clinical outcomes [14]. Since our study did not have a long-term follow-up, further research will be needed to confirm the long-term prognosis of each treatment. In addition, it is necessary to determine the radiological outcomes according to differences in treatment methods.

There are several limitations of the current analysis. First, although a satisfactory number of studies was included, the number of RCTs was small. Pooling the results of retrospective studies may lead to an overestimation of the outcomes. Nevertheless, since no publication bias was observed in the present meta-analysis, our results were meaningful. Second, we could not evaluate the risk of bias arising from the variable quality of different arthroplasty systems and concepts; this is inevitable when performing a synthetic study. Third, we did not include any newly invented or modified devices or systems. Further studies that include high-quality trials of new models are needed. Finally, considering the clinical significance of arthroplasty, the mean follow-up of the studies included in our analysis was short. Although we did not find any difference in revision rate between the systems, future studies comparing long-term outcomes are needed.

## Conclusions

The results of our meta-analysis showed that a larger ROM can be achieved with aTSA than with rTSA, but they did not differ in revision rate at the mid-term follow-up. Glenoid loosening was more common with aTSA than with rTSA, and scapula notching was more common with rTSA, as per radiological findings. Considering our results, surgeons should carefully select the treatment option while keeping in mind the patient’s needs and functional activity when treating primary glenohumeral arthritis.

## Data Availability

Not applicable.
